# Developmental variations in the impact of intimate partner violence exposure during childhood

**DOI:** 10.5249/jivr.v8i1.663

**Published:** 2016-01

**Authors:** Kathryn H. Howell, Sarah E. Barnes, Laura E. Miller, Sandra A. Graham-Bermann

**Affiliations:** ^*a*^Department of Psychology, University of Memphis, Memphis, US.; ^*b*^Psychology and Peace Studies, University of Notre Dame, Notre Dame, US.; ^*c*^Department of Psychology, University of Michigan, Ann Arbor, US.

**Keywords:** Domestic violence, IPV, Witnessed violence, Children, Review

## Abstract

**Background:**

Intimate partner violence (IPV) is a pervasive problem impacting individuals around the globe. The consequences of IPV extend beyond the adults in the relationship, as children witness a significant proportion of such violence. Exposure to IPV during childhood has devastating effects across multiple domains of functioning.

**Methods:**

This article reviews empirical studies of the effects of exposure to IPV by developmental stage.

**Results:**

The psychological, social, physical, and cognitive consequences of witnessing IPV are examined across development; from the impact of prenatal exposure to effects in infancy and toddlerhood, the preschool years, school-aged children, and adolescence.

**Conclusions:**

The review concludes by providing suggestions for future research based on the identified developmental variations, recommendations for developmentally-sensitive interventions for children who have witnessed IPV, and directions for policy to address the issue of violence exposure early in the lives of children.

## Introduction

Approximately 30% of women in the United States are exposed to mild intimate partner violence (IPV) in their lifetime, defined as being pushed, shoved or slapped, and about 25% are exposed to severe IPV, which includes being beaten, burned, choked, and/or violence involving a weapon.^1^ When evaluated worldwide, the prevalence of IPV shows significant variability, with yearly rates of physical and sexual violence as low as 3% in Serbia and Montenegro to 54% in Ethiopia.^2^ Violence in a partnership has more extended effects on the family system, as children are eye-witness to approximately 80-95% of such aggression.^3-4^ In the United States alone, more than 1 in 15 children witness IPV each year^5^ and worldwide estimates indicate that approximately 275 million children are exposed to IPV on a yearly basis.^6^

For more than 25 years, researchers from around the world have studied the impact of IPV on diverse areas of children’s development, including emotional and behavioral adjustment, cognitive functioning, structural brain development, school performance, and physical health.^7-17^ Several studies have identified profiles of adjustment for children exposed to IPV and find that about 35% are in the clinical range on internalizing and externalizing adjustment problems, 45% are in the borderline range on adjustment problems and about 20% experience low or no adjustment problems, suggesting some resilience.^18-19^ Studies of long term effects have shown that childhood exposure to IPV is associated with increased risk for delinquency, greater mental health problems, and the potential for intergenerational violence in dating and intimate partner relationships.^20-24^

The problem is extensive, the stakes are high, and yet the solutions are just beginning to be theorized and empirically evaluated. In this review, we describe the current research on outcomes associated with exposure to IPV for children at different developmental stages to underscore the intersection of developmental and environmental variables. We conclude with recommendations for future studies, developmentally sensitive interventions, and the need for policy to address the issue of IPV early in the lives of children.

**Prenatal through Toddlerhood (0-2 years)**

The prevalence of IPV during pregnancy has been a primary research focus in recent decades, with many studies reporting that pregnant women are at an increased risk for experiencing violence as compared to non-pregnant women.^25^ Numerous methodological issues, however, have made it difficult to draw clear conclusions about IPV-risk and pregnancy. Nevertheless, the prevalence of IPV around and during pregnancy is alarming, with rates as high as 36% depending on the sample and the form of violence examined.^26^ This rate of violence during the 9-10 months of pregnancy is especially concerning because it parallels women’s lifetime likelihood of violent experiences. Given that violent relationships typically last for many years, the span of exposure likely places children at risk from the prenatal period and into infancy and toddlerhood.^27^ This is confirmed by statistics from the National Survey of Children’s Exposure to Violence, in which it was shown that witnessing a familial assault was one of the most commonly experienced forms of violence during infancy.^5^ Additionally, Graham-Bermann and Perkins found that 64% of children exposed to IPV initially witnessed this violence in their first year of life.^28^ Thus, the adverse consequences associated with early IPV exposure set the stage for continued difficulties throughout infancy and toddlerhood. 

**Social and Emotional Functioning **

Children’s functioning is indirectly impacted by IPV exposure prenatally, in part because of the mental health consequences experienced by their mothers. Mother’s level of distress during pregnancy affects parental warmth, caregiving and the development of healthy attachment patterns. ^29-30^ Unfortunately, these early and serious risks to fetal development do not necessarily abate following birth. Continued IPV exposure likely interferes with infants’ and toddlers’ attachment relationships, particularly with their mother. For example, in a study of 72 mothers and their 15-month old infants, mother’s IPV exposure was associated with her child’s disorganized attachment style, with nearly two-thirds of the infants classified as insecurely attached.^29^ Only one third of infants had secure attachments and these infants had mothers who experienced less severe violence. Longitudinal research indicates that IPV is related to children’s attachment to their mother over time, with lower levels of IPV predicting more stable attachment relationships and increases in IPV disrupting secure attachments.^30^ Thus, the threat that IPV poses to primary caregiving relationships and social development during infancy is serious and linked with prolonged difficulties across childhood.^30^

In terms of the emotional toll that IPV exposure takes on infants and toddlers, a large scale study by Lundy & Grossman analyzed data on over 40,000 children aged 1-12 collected over a five-year period from approximately 50 domestic violence agencies.^31^ In their sample of approximately 13,000 1-2 year old children, the researchers found that nearly 38% had one or more problems with emotion regulation, two thirds were struggling to separate from a parent, and nearly 42% were crying often. Another study examined infants’ and toddlers’ emotional difficulties by having their mothers report on their temperament.^32^ Results indicated that mothers who were current victims of IPV were more likely to report that their child had a difficult temperament, characterized by fussy, irritable behavior and difficulty soothing. 

**Psychological and Behavioral Functioning**

Multiple studies have documented increased adjustment problems, including internalizing and externalizing behaviors, in infants and toddlers who live in homes with IPV. For example, one study examined the relationship between exposure to IPV, nonviolent angry verbal conflict, and adjustment difficulties for 1-3 year old children.^33^ Results revealed that children exposed to IPV had significantly higher levels of adjustment problems, particularly in regards to atypical or maladaptive behaviors (e.g., making odd sounds, repetitive movements).Another study found direct relationships between mother’s exposure to IPV prior to and during pregnancy and infant’s externalizing behavior problems, a relationship that was mediated by parenting practices.^34^ Adjustment problems have also been examined longitudinally, with research indicating that relative to nonexposed children, children who had directly witnessed IPV were 2.72 and 3.48 times more likely to have externalizing symptoms above the clinical cutoff at ages 2 and 3, respectively.^35^ Findings for internalizing behaviors were not significant, though the authors noted internalizing symptoms may be more subtle and easily missed during this developmental stage. 

With regard to specific forms of psychopathology, exposure to IPV has been linked to the presence of posttraumatic stress symptoms, even in infancy.^36^ In their study of 48 mothers and their 1-year old infants, Bogat and colleagues reported that nearly half (44%) of the infants exhibited at least one trauma symptom.^36^ Additionally, researchers identified a significant relationship between maternal and infant trauma symptoms, such that when mothers experienced severe IPV and displayed evidence of posttraumatic stress, so did their infants. This finding suggests that the impact of violence exposure may be transactional for young children, who have a combined burden of distress as they contend with their individual reaction to IPV and that of their parent’s.^37^

**Physiological Functioning and Physical Health Problems**

The physical health ramifications for children exposed to violence during this developmental period are immense, with IPV during pregnancy linked to heightened risk for premature birth, low birth weight and use of intensive care services.^38-39^ Exposure to IPV during pregnancy is also associated with increased risk for fetal death, miscarriage, and maternal mortality.^40^ In examining the physical health consequences of IPV exposure during the early years of life, Lundy and Grossman found that nearly 10% of 1-2 year olds had at least one physical health problem, the most common being reports of frequent illness (45.3%).^31^ In terms of specific health outcomes, toddlers in families characterized by chronic IPV were twice as likely to be diagnosed with asthma as compared to children from families without such violence.^15^

Beyond physical health consequences, the impact of IPV exposure on the physiology of young children has recently garnered significant attention. In a study of cortisol reactivity in infants, Hibel, Granger, Blair, and Cox examined adrenocortical dysregulation in toddlers who had been exposed to high and chronic levels of IPV. ^41^ Cortisol dysregulation was present at age 24-months, but not present when children were 7- and 15-months old, suggesting that dysregulation may be a product of chronic adaptation to stress. Similarly, Sturge-Apple, Davies, Cicchetti, and Manning identified low cortisol reactivity in the infants of women who both experienced IPV and were emotionally unavailable.^42^ Continued work on the relation between IPV-exposure and children’s physiology during this developmental period is called for as dysregulation in the stress-response system may increase the risk for difficulties later in life. 

**Cognitive and Intellectual Functioning**

Research documenting the cognitive and intellectual functioning of infants and toddlers exposed to IPV is extremely limited. Given that the first 12 months of life may be a particularly critical time in cognitive and social development,^43^ more research focusing on infants and toddlers exposed to IPV is needed. One of the few articles to document cognitive functioning in the context of early IPV-exposure used longitudinal data to assess IPV exposure at ages 2-4 and then linked this exposure with later school engagement in middle childhood. ^44^ Results showed that 35% of mothers of 2- to 4-year olds reported IPV in the past 12 months and 16% reported an increase in victimization over time. Interestingly, the authors found that the increase of IPV, but not early IPV exposure, significantly predicted decreased school engagement in middle childhood. This finding suggests that continued, chronic exposure is more influential than single shorter episodes of violence. There is also evidence suggesting that increased attention to threatening stimuli during this time period may place children at risk for the development of internalizing problems, including social withdrawal and anxiety.^45-47^

The body of literature documenting the impact of IPV on infants and toddlers is relatively small compared to that for older children. However, the significant challenges that such exposure pose to children during this developmental period require that more extensive research be conducted. As such, future research must focus more closely on this foundational stage to further understand the short and long-term effects of IPV across domains of functioning. 

**Preschool-Aged (3-6 years)**

Children in the preschool-age range have one of the highest rates of exposure to IPV of any age group and research supports a diverse range of detrimental effects resulting from such exposure.^48^ Especially important to consider for preschoolers is the extent to which they may rely on parents and primary caregivers for both basic needs (such as safety) and modeling for more advanced processes (such as emotion regulation). Also unique to this age range is the burgeoning sophistication with which young children can report on their internal cognitions.^49^ A key consideration for preschoolers’ functioning during these years is the fact that most of them begin to inhabit at least two environmental contexts (home and preschool or daycare). Researchers have demonstrated that risk and protective factors in both domains are highly influential in children’s development, but there is also an indication that risk in one domain may result in decreased competencies in another, which highlights the need for a dimensional and interactional approach to understanding preschoolers’ functioning after exposure to IPV.^50^

**Social and Emotional Functioning**

As preschoolers begin to venture outside of the home, healthy and prosocial interactions with peers is one marker of success. Those children who have secure attachments with primary caregivers exhibit the social and emotional strengths that are necessary to engage effectively with peers.^51^ For children exposed to IPV, however, social competence may be reduced, which impairs the extent to which children are able to develop healthy relationships with peers and others outside of the home.^52^

Just as children may use the parental relationship as a secure base to develop their relationships with others, preschoolers frequently look to primary caregivers to establish important self-regulatory skills.^53^ When children are chronically exposed to intimate partner violence, emotion regulation difficulties consistently emerge.^54-55^ Dysregulated children may exhibit aggressive behaviors toward siblings,^54^ peers^56^ or parents and authority figures.^57^

**Psychological and Behavioral Functioning**

Beyond impairment in children’s social and emotional functioning, exposure to IPV has insidious effects on pre-schoolers’ psychological and behavioral health. Most recent research on IPV exposure in preschoolers has recognized that posttraumatic stress symptoms and posttraumatic stress disorder (PTSD) are evident, even in this young population. Based on new DSM-5 criteria for PTSD in children ages six and under,^58^ it is possible that upwards of 50% of preschoolers experience clinically significant symptoms of PTSD following exposure to IPV.^9^ Preschool-onset PTSD has also been linked to higher threat attenuation in young children exposed to IPV.^59^ It may be that during the preschool years, children become more developmentally attuned to threat in general, and the presence of family violence during these years may pose a particularly serious risk for the maladaptation of threat detection systems, possibly explaining the early development of PTSD and anxiety in this age group.^47^

Meta-analyses focusing on child internalizing and externalizing problems suggest discrepant outcomes regarding the relative risk for preschool children, with some showing no differences in risk by age^7^ and others identifying heightened risk in the preschool years.^16^ The latter of these studies, however, notes that while psychological problems are prevalent in children exposed to IPV, there are too few studies and too great a disparity in outcome to draw clear conclusions regarding age-based risk.^16^ Generally speaking, there is a great need for longitudinal research that follows children for longer periods of time in order to better establish patterns of psychopathology across childhood.

**Physiological Functioning and Physical Health Problems**

In recent years, physiological effects of exposure to IPV during the preschool years have been examined as possible explanatory mechanisms by which social, emotional and behavioral problems emerge. Research in this domain has ranged from the examination of the sympathetic and parasympathetic nervous system^60^ to glucocorticoid responses.^61^ This research has demonstrated that preschool children exposed to IPV may have heightened heart rate and cortisol response patterns compared to their non-exposed peers.^61^ The relationships between physiological functioning and emotional and behavioral functioning, however, are complex and one recent study showed that for children living in highly violent contexts, parasympathetic functioning was unrelated to parents’ reports of preschoolers’ difficulties. ^60^ In all, there is a dearth of research on physiological effects of IPV and the direction and magnitude of these effects on functioning for preschool-aged children.

Although there is a lack of research on physiological processes, a number of recent studies have examined the effects of IPV on preschoolers’ physical health. Preschool children exposed to IPV appear to be at particular risk for early onset obesity, even after controlling for other relevant risk factors.^62^ The pathways of risk for obesity in these children are complex, but could include impaired regulatory mechanisms,^53^ as well as neighborhood and family level risk factors.^62^ IPV appears to contribute to health problems in a dose-response pattern.^11^ Although total health problems are linked to higher rates of PTSD in large-scale studies,^11^ this has not been replicated in smaller samples.^63^ This study, however, did find that gastrointestinal problems, and specifically asthma, were related to higher risk for adjustment problems in preschoolers who witnessed violence.^63^

**Cognitive and Intellectual Functioning**

The extent to which IPV affects preschoolers’ cognitive and intellectual functioning may be quite profound, especially in light of the transition to school contexts during this period. Research on preschoolers’ verbal ability has shown that those exposed to violence have suppressed IQ’s as compared to same-age peers.^64^ IPV severity has also been linked to poorer memory functioning in preschool children.^65^ Across studies, cognitive and intellectual abilities are influenced by other environmental and contextual factors that may be impacted by the presence of IPV in the home, such as maternal education, socioeconomic status, maternal depression, and parenting.^64-66^ Available research on preschool children exposed to IPV highlights the multitude of difficulties associated with witnessing such violence during this developmental stage. Given that preschoolers experience some of the highest rates of violence exposure, likely because they spend a significant amount of time in the home and in the presence of their caregivers, they require close attention from researchers and clinicians alike. 

**School-Aged (6-12 years old)**

As children progress into the school-age years, they develop a more sophisticated understanding of themselves and others, including more complex thought patterns and unique worldviews.^67^ This developmental period also marks an important shift into the formal school system, which brings about a host of influences beyond the family, including peer and social groups that play an increasingly important role in children’s developing self-concept.^68^

**Social and Emotional Functioning**

Recent work has shown that many school-aged children who witness IPV have difficulties in developing and maintaining friendships, as well as an increased likelihood of developing maladaptive peer relations.^31,69^ For example, in a sample of 16, 467 violence-exposed school-aged children, more than half had one or more social problems, such as being overprotective of family members, engaging in parent-child role reversal, and behaving like a younger child.^31^ Children who witness violence also report higher levels of conflict with a close friend and endorse feeling lonelier than their non-exposed peers.^69^ Such findings indicate that the process of connecting with peers and developing healthy friendships is hindered in school-aged children who witness IPV, which may lead to ongoing difficulties with peers, including bullying and victimization.

Research on the relationship between exposure to IPV and bullying and victimization at school has been assessed in a sample of Italian children (mean age was 11.2 years), with findings indicating that exposure to IPV significantly predicted school bullying and victimization, even after controlling for direct child abuse.^70^ In a similar study, Bauer, Herrenkohl, Lozano, Rivara, Hill and Hawkins examined 112 children aged 6-13 who completed assessments of their own experiences of bullying or victimization within the past year, while parents reported on the child’s internalizing and externalizing symptoms and their own relationship violence^71^ Unlike the former study,^70^ exposure to IPV was not significantly associated with bullying or victimization by peers, despite a high prevalence of both (33.9% and 73.2%, respectively). While these inconsistent findings call for more empirical research, existing studies suggest that difficulties in social dynamics may be linked to maladaptive attitudes about violence. For example, Graham-Bermann and Brescoll’s research on stereotyped beliefs in school-aged children indicated that witnessing inter-parental violence was associated with more rigid conceptions of gender roles and greater acceptability of violence, both of which have implications for children’s interactional style and ability to relate to peers.^72^

With regard to emotional functioning, Katz, Hessler and Annest longitudinally examined the influential role of emotional competence in promoting IPV-exposed children’s peer relations.^73^ IPV exposure was assessed when children were 5-years old, emotional competence was assessed at 9.5-years and peer relations and behavioral adjustment were measured at age 11. Emotional competence mediated the relation between witnessing IPV and children’s peer relations and adjustment. Children with poor emotional competence were less aware of their emotions, showed more difficulty regulating their emotions, and had more negative peer interactions and social problems at age 11. These children also had significantly more behavior problems at the final assessment. 

**Psychological and Behavioral Functioning**

The psychological and behavioral functioning of school-aged children who witness IPV has received considerable attention to date. Witnessing IPV greatly affects school-aged children’s behavior, particularly with respect to internalizing and externalizing problems.^74-75^ These behavioral difficulties are common in violence-exposed school-aged children, as evidenced by a recent meta-analysis that found the mean weighted effect size for internalizing problems was d = .51 and for externalizing problems was d = .49, indicating moderate effect sizes for both sets of problems.^7^ Lundy & Grossman found that over 58% of violence-exposed school-aged children had one or more psychological difficulties connected with witnessing violence in the home.^31^ These children were more likely to experience mood swings, to be afraid, and to resist interacting with others.^31^

In regards to more specific effects of witnessing IPV, the relationship between violence exposure and mental health was examined in a large and representative sample of children in Great Britain.^76^ This study indicated that exposure to IPV nearly tripled the likelihood of children having conduct disorder, but was not independently associated with other disorders. One mental health outcome that has garnered significant attention is posttraumatic stress. Graham-Bermann and Levendosky examined posttraumatic stress symptoms in a sample of IPV-exposed and non-exposed children and found that slightly more than half of the children who witnessed IPV suffered from intrusive thoughts regarding the exposure and 42% exhibited symptoms of hyperarousal.^74^ Further, almost one fifth of violence-exposed children displayed avoidance of the trauma, and 13% qualified for a full diagnosis of PTSD. Chemtob and Carlsonalso studied posttraumatic stress symptoms in 25 mother-child dyads (mean child age was 11.2) in which the mother experienced IPV.^77^ Even though the violent relationship had ended, on average, more than two years prior to study participation, 40% of children reported posttraumatic stress symptoms that warranted a diagnosis of PTSD. Additionally, children’s posttraumatic stress symptoms were significantly and positively correlated with their anger, as evidenced by frequent outbursts of anger both at home and in the community. While this study is limited by a small sample size, it is noteworthy in that it highlights the difficulties children experience even after multiple years have passed since the violence ended.

Symptoms of depression and anxiety have also been studied in relation to posttraumatic stress among children exposed to IPV. Reynolds, Wallace, Hill, Weist and Nabors’ research revealed that for IPV-exposed boys, but not girls, higher levels of posttraumatic stress symptoms were associated with higher levels of depression and lower self-esteem.^78^ Kennedy, Bybee, Sullivan and Greeson measured symptoms of anxiety and depression over a period of two years, with results showing that IPV exposure was associated with high levels of depression and anxiety, initially, but that this relationship decreased as time progressed.^12,79^ In a longitudinal assessment of the association between violence exposure and mental health outcomes, Johnsona and colleagues found that witnessing family violence significantly predicted higher levels of depression, anger, and anxiety in 6-8 year old children.^80^ Additionally, the levels of these outcomes increased linearly as the amount and severity of the violence increased.^80^

**Physiological Functioning and Physical Health Problems**

In addition to psychological and behavioral problems following school-aged children’s violence exposure, recent work has uncovered physical and physiological consequences as well. El-Sheikh and Harger examined the relation between children’s appraisals (e.g., self-blame and perceived threat) of marital conflict and their adjustment, physical health, and physiological reactivity.^81^ Heart rate, systolic and diastolic blood pressure, and skin conductance were measured in 89 elementary-aged children during both a baseline and experimental session in which children listened to a recording of an adult argument. Results revealed that children’s appraisals of self-blame and threat moderated their physical health and cardiovascular reactivity, as well as their internalizing and externalizing behavior problems. Authors interpreted these results as evidence of a robust vulnerability factor associated with children’s appraisals and stated that such appraisals may serve to activate stress responses. 

Recently, there has been an interest in the role of the vagus nerve in regulating emotion and influencing the physical health of IPV-exposed children. Identified as one marker of physiological regulation, vagal tone is an indication of the influence of the parasympathetic nervous system on the heart, with increases in vagal tone slowing heart rate and decreases in vagal tone increasing heart rate.^82^ Katz examined whether individual differences in children’s vagal reactivity were related to exposure to IPV and found that children with conduct problems who showed vagal augmentation to a peer provocation came from families with high levels of IPV.^83^ It has been suggested that vagal augmentation in children from violent homes has an adaptive function, in that children living in such environments may need to closely monitor their surroundings for signs of threat or interpersonal negativity.^84^

Links between children’s physica health and IPV exposure are also evident. Lundy & Grossman reported that 27% of children age 6-12 had at least one physical health problem, with 35.8% of these children reporting bed wetting, 23.8% frequent illness, and 22.6% weight problems.^31^ Another study examined the physical health of IPV-exposed children age 6-12 by using the somatic items from the Child Behavior Checklist^85^ to compare the health of exposed and non-exposed children in The Netherlands.^86^ Results revealed that IPV-exposed children more often experienced health complaints, particularly in the domains of eating, sleeping, and pain. Odds ratios ranged from 2.05-30.01, with eight items (constipation, nausea, overtired, trouble sleeping, nightmares, aches/pains, stomachaches, and dizziness) having an odds ratio higher than five. These results suggest that witnessing IPV places a considerably large physiological and physical health burden on school-aged children.

**Cognitive and Intellectual Functioning**

The impact of witnessing IPV on children’s cognitive and intellectual functioning has also garnered attention from researchers. Such research links the emotional and behavioral difficulties of children exposed to IPV to deficits in executive functioning (EF), which reflects difficulties with planning, prioritizing, organizing, and task completion.^87^ DePrince and colleagues compared EF in children who were exposed to family trauma (e.g., witnessing IPV or physical or sexual maltreatment), to non-familial traumas (e.g., motor vehicle accident), or to no trauma exposure.^87^ Findings indicated that there was a medium effect size for the relationship between family-based trauma and EF deficits. Interestingly, this effect remained significant even after controlling for trauma-relevant cues, suggesting that children exposed to IPV show poorer EF performance than their non-exposed peers, even in the absence of emotional content related to the trauma.

Children’s academic progress has also been examined with regard to IPV exposure. Lundy & Grossman found that more than one fifth (21.6%) of children age 5-12 who witnessed IPV had one or more educational difficulties, with nearly half (47.2%) of those children experiencing a specific learning problem.^31^ In terms of standardized test score performance, one study indicated that school-aged children residing in homes with IPV had significantly lower performance on standardized math, reading, language, and core test scores than children without IPV in their home.^88^ For school-aged children, these differences ranged from 17.3 points (math) to 20.4 points (both core and language) and constituted the largest discrepancies between exposed and non-exposed children. These results suggest that IPV exposure is linked to significant academic problems for children that are likely to carry over across the years and impede future academic success.

**Adolescence (13-18 years old)**

Adolescence marks a period that is characterized by identity formation and the solidification of a working self-concept.^89-90^ For many, this stage also involves a burgeoning sense of autonomy and expansion of social relationships. Thus, by adolescence, the consequences of witnessing IPV clearly extend beyond the boundary of the family.^67^ Additionally, adolescence is associated with increased risk for experiencing other forms of violence, both indirectly and directly.^5^ Given the immense developmental tasks of this period as well as other potential risks posed by the environment, it is important to understand how IPV uniquely affects adolescents across domains of functioning.

**Social and Emotional Functioning**

One primary social implication of IPV exposure is emerging dating violence during adolescence. Lichter and McCloskey examined the role that attitudes surrounding violence perpetration play in dating violence and found that adolescents who grew up in homes characterized by IPV were more likely to develop attitudes that accepted violence as a viable means of conflict resolution, which were in turn associated with higher levels of dating violence perpetration.^91^ Similarly, Temple, Shorey, Tortolero, Wolfe and Stuart evaluated attitudes about violence as well as the gender of the perpetrator.^92^ Results indicated that for girls, both mother- and father-perpetrated IPV was associated with increased psychological and physical dating violence perpetration, whereas only mother-perpetrated IPV was associated with physical dating violence for boys. Further, these relationships were fully mediated by attitudes that were accepting of female violence (for girls) and male violence (for boys). Both of these studies highlight the important role that attitudes surrounding violence play in perpetration of later IPV.

With regard to emotionality, significant aggressive behavior has been documented in adolescents following IPV exposure. For example, Haj-Yahia and Abdo-Kaloti showed that witnessing IPV significantly predicted aggression in adolescents from Palestine,^93^ and Ghasemi identified higher levels of hostility among IPV-exposed Iranian youth.^94^ Similarly, McCloskey and Lichter found that adolescents exposed to family violence were more aggressive with their peers, and, in some cases, dating partners and parents.^95^ In an examination of bullying and victimization among adolescents, the research of Knous-Westfall, Ehrensaft, MacDonnel and Cohen indicated that IPV exposure predicted higher levels of overt peer victimization in adolescents.^96^ When IPV exposure was high, significant associations were found between adolescent’s relational bullying and their own overt victimization. Gender differences in victimization and bullying also emerged, with girls experiencing higher levels of relational peer victimization when any IPV was reported, and boys presenting with higher levels of overt peer bullying when severe IPV was present in the home.

**Psychological and Behavioral Functioning**

As is consistent with other developmental stages, witnessing IPV plays a prominent role in adolescents’ problematic psychological functioning. A number of studies have examined the relation between IPV exposure and adolescents’ reports of anxiety and depression. For instance, Haj-yahia and Abdo-Kaloti found that family violence significantly predicted Palestinian adolescents’ anxiety, depression, and withdrawal even after controlling for relevant sociodemographic factors.^93^ Similarly, a nationally representative study of Danish youth showed that exposure to violence within the home was significantly associated with higher reports of anxiety and depressive symptoms.^97^ In a sample of Filipino adolescents, the research of Hindin and Gultiano revealed that among both male and female youth, exposure to IPV was strongly predictive of increased frequency of depressive symptoms.^98^

Delinquency and other high risk behaviors have also been studied in this population. In regards to the former, Herrera and McCloskey reviewed juvenile court records of a sample of adolescents who were previously interviewed about family violence and found that IPV exposure predicted court referrals for both males and females.^99^ Using a large community sample of adolescents, Ireland and Smith examined the relation between IPV-exposure and both current and later antisocial behavior and relationship violence.^100^ After controlling for a number of relevant factors, IPV exposure significantly predicted antisocial behavior in adolescents, risk for being in a violent relationship and committing violent crime in young adulthood.

The association between witnessing IPV and the prevalence of posttraumatic stress disorder and major depressive episodes has also been assessed in a nationally representative sample of U.S. adolescents who witnessed IPV.^17^ These adolescents were twice as likely to have PTSD and 1.7 times as likely to have had a major depressive episode within the last six months. Notably, the associations held after controlling for key sociodemographic variables and direct victimization experiences. Ghasemi found that Iranian youth exposed to IPV had significantly more trauma symptoms than youth not exposed to family violence.^94^ In a study examining relations between exposure to IPV, PTSD, and aggression, Moretti, Obsuth, Odgers and Reebye indicated that adolescents with a diagnosis of PTSD were more aggressive than adolescents without a diagnosis, suggesting that mental health outcomes impact youth’s functioning across psychological domains.^101^

Like children in younger age groups, adolescents exposed to IPV generally exhibit problematic levels of internalizing and externalizing symptoms. In a meta-analysis by Evans and colleagues, mean weighted effect sizes for adolescent internalizing and externalizing symptoms were .51 and .40, respectively, suggesting moderate difficulties for youth in this age bracket.^7^ A mega-analysis by Sternberg, Baradaran, Abbott, Lamb, and Guterman revealed that IPV-exposed early adolescents were 4.9 and 2.1 times more likely than their non-exposed peers to reach the clinical cutoff on externalizing and internalizing behaviors, respectively.^102^ A number of studies have examined more nuanced variables related to IPV that might influence the expression of internalizing and externalizing symptoms during adolescence. For example, Wright and Fagan assessed whether the gender of the perpetrator of family violence influenced the presentation of symptoms.^103^ They showed that IPV perpetrated by females predicted increased internalizing problems in girls, but not boys, even after controlling for a number of relevant variables (e.g. other demographic factors, history of child abuse). Interestingly, this pattern of results was not seen for male-only perpetrators or mutual violence between both caregivers, suggesting that the gender of the perpetrator plays a strong role in outcomes during adolescence. Another study took a more dimensional approach to assessing family violence and examined whether an IPV index (comprised of frequency, proximity, and duration of IPV) predicted adolescent’s psychosocial problems above and beyond occurrence alone.^104^ While concerns over multicollinearity prevented an examination of each dimension individually, this indexed approach predicted youth’s self-reported internalizing and externalizing symptoms above the presence of IPV.

**Physiological Functioning and Physical Health Problems**

In regards to the physical effects of IPV exposure on adolescents, many studies report general somatic problems, rather than specific outcomes. For example, in a large sample of Palestinian adolescents, Haj-yahia and Abdo-Kaloti found that adolescent’s exposure to family violence significantly predicted their somatization complaints, above and beyond relevant sociodemographic characteristics.^93^ Similarly, Ghasemi established that somatic symptoms were significantly higher in Iranian youth who had been exposed to family violence relative to non-exposed adolescents.^94^ Lastly, in a study of Finnish adolescents, Lepisto, Joronen, Astedt-Kurki, Luukkaala, and Paavilainen showed that adolescents from homes with IPV rated their physical health significantly lower than youth from nonviolent homes.^105^

One study conducted in the United States evaluated trajectories of BMI in a group of children and adolescents (age 9-14 at baseline) followed from 1996-2004.^106^ Using general growth mixture modeling, researchers found that boys exposed to IPV early in life (ages 0-5) were more likely to be obese or steadily overweight in late adolescence, while boys exposed to violence in middle childhood (6-11 years) were more likely to be obese. For girls, early exposure to violence (ages 0-5) was associated with an elevated risk for being steadily overweight in late adolescence. This study is noteworthy in that it outlines the long-term physical health consequences of IPV exposure that carry forward from childhood into adolescence.

**Cognitive and Intellectual Functioning**

Only a handful of studies have examined the influence of IPV-exposure on adolescent’s cognitive and intellectual functioning. Within this small literature, the impact of IPV on autobiographical memory has been of particular interest. One study longitudinally examined recollections of family violence by asking adolescents (mean age of 15) about documented events 6 years earlier.^107^ Findings revealed that many of the participants neglected to report key details of events, with more than one third of adolescents failing to remember or acknowledge witnessing IPV. 

Using the same sample of adolescents, Johnson, Greenhoot, Glisky, and McCloskey examined the relation of IPV exposure, adolescent’s depressive symptoms, and autobiographical memory for childhood experiences.^108^ Findings indicated that adolescents exposed to more recent episodes of family violence had more memory problems, such as overgeneralized memories, shorter narratives, and fewer negative memories, with self-reports of depression predicting only overgeneralized memories. An earlier study of adolescent’s memory retrieval found that only high levels of self-reported depression predicted overgeneralized memories, not family violence exposure.^109^ More work in this area is needed to further delineate the impact of IPV-exposure on later memory performance, as this may be a central cognitive process that impacts functioning following violence exposure.

In regards to academic performance, Jayasinghe, Jayawardena and Perera found that IPV exposure was significantly associated with Sri Lankan adolescents’ school performance and attendance, in that adolescents from violent homes were 2.8 times more likely to have failing grades and 3.8 times more likely to attend school less than 80% of the time, as compared to their non-exposed age mates.^110^ In an examination of the relation between IPV and standardized test scores, Peek-Asa and colleagues found no significant difference in the performance of IPV-exposed and non-exposed adolescents.^88^ The discrepant findings from these studies highlight the need for more work in the domain of adolescent’s academic performance.

In sum, research on the impact of IPV exposure during adolescence highlights the vast and chronic challenges faced by individuals during this developmental stage. Of utmost importance is the ongoing cycle of violence that emerges during this developmental period, with increases both in dating violence and peer aggression for adolescents who witness violence in the home. Such findings underscore the need for early intervention and treatment to curb the perpetuation of violence.

## Conclusion

**Common Outcomes**

It is clear that exposure to IPV places a great burden on children across developmental stages and that problematic outcomes are noted in children from a range of cultural and socioeconomic backgrounds across the world. Interestingly, the concerning consequences of witnessing IPV appear to place children at a similar burden of risk across countries, with evidence of psychological, physical, and social ramifications in disparate regions from Palestine to the Netherlands. The effects can be seen prenatally and continue through adolescence, with adjustment and mental health challenges documented as early as infancy. One of the greatest difficulties falls within the social domain, as youth across ages have problems in relationships with others, from attachment bonds in infancy to making friends in school to navigating healthy dating relationships during adolescence. This review also highlights that exposure to IPV challenges children’s developing cognitive abilities, their executive functioning, and therefore their academic performance, all of which may lead to problems obtaining fruitful advanced education and job success. Finally, the studies we have examined suggest that witnessing IPV places a large physiological burden on children, regardless of their age. With more illness, less physical fitness and reduced ability to regulate emotions, these children may not progress optimally into adulthood. 

**Unique Age-Related Issues**

While there are common challenges across developmental stages, there are also unique issues that face children at specific ages. For instance, infants may be born early or with low birth weight, which impairs healthy attachment and possibly sets off a cascade of developmental dysregulation that could last a lifetime.^38-39^ By toddlerhood, self-regulatory abilities are challenged and externalizing problems are prominent.^35^ Yet, it is during the preschool years that children are most likely to witness IPV.^48^ For some young children, the consequences of IPV exposure during this stage heavily impact social development. Further, effects on physical health beyond early low-birth weight begin to emerge during the preschool years, with studies documenting greater obesity, asthma and gastrointestinal problems for exposed compared to non-exposed preschool age children.^62,60,11,63^

When children enter the formal school system, they are influenced by those outside of the family to a greater extent than ever before. Many school-aged children exposed to IPV develop poor or maladaptive peer relationships.^31,69^ Their levels of traumatic stress and other adjustment difficulties may further inhibit their ability to develop close friendships, which is a hallmark of success during this stage. The wave of negative effects continue into adolescence, where researchers have identified the emerging cycle of violence as adolescents perpetuate aggression in their burgeoning dating relationships, likely in part linked to the maladaptive perspective that violence is justified and acceptable.^91^

**Controversies and Inadequate Information**

While there has been a great deal of attention given to the topic of how IPV affects children, more scholarship is needed. This is especially evident with regard to research evaluating IPV across countries. While this review highlights the multitude of consequences faced by children around the world who have witnessed IPV, there is a great need for comparative research on possible variations in the nature of IPV and the expression of consequences of violence exposure based on cultural, regional, or political differences that have not yet been empirically examined. For example, future scholarship could evaluate culture-specific outcomes, how legal and policy efforts related to IPV vary by country, or how researchers can best tailor interventions and resources to be culturally-sensitive.

A number of additional areas for future study can be identified by research that is inconclusive or contradictory. One overarching issue is whether there is an age-related risk for developing psychological and adjustment problems. For example, psychopathology is prevalent in children exposed to IPV, but there are too few longitudinal studies and too much variability in outcomes to make any conclusive statements regarding trajectories of risk related to age. The area of physiological functioning is also relatively understudied and there are divergent findings in regard to how IPV affects functioning in this domain. For example, some studies find a link between parasympathetic system functioning and children’s behavioral problems following violence exposure, while others do not find such an association. Research on cortisol stress reactivity also needs further documentation to develop a body of consistent findings that show the impact of IPV exposure on physiology. Lastly, more in depth and sophisticated studies on the cycle of violence are urgently needed. Longitudinal research that documents how attitudes and beliefs about violence developed during the early childhood years contribute to later peer bullying, victimization, and dating violence will help to identify key times for intervention. Beyond examining pathways of risk, there is a strong need for additional research on pathways of resilience and positive functioning in children exposed to IPV, including an assessment of protective factors and variables that may promote adaptive development. While some researchers have begun to explore this topic,^111,112^ there remains a dearth of information on how to best identify and promote resilient functioning among violence exposed children. 

**Recommendations for Early Intervention**

It is clear that the effects of IPV on children are evident and influential even in-utero. The costs are substantial, with cascading effects that last far into adulthood. To address these deleterious consequences, clinicians, researchers and policy makers must consider the needs of pregnant women, the needs of children living in the home, and the needs of adolescents and young adults as they transition into new formative relationships. Without intervention, the effects of violence exposure will likely grow exponentially as children move from one age and stage to the next. Thus, by providing early detection and intervention, we can, perhaps, break the cycle of violence and prevent future generations of children from being exposed to the epidemic of intimate partner violence.
